# Financial resources, access to care, and quality of care mediate racial disparities in statin usage for secondary prevention

**DOI:** 10.1371/journal.pone.0311724

**Published:** 2024-10-08

**Authors:** Christopher Wong, Lyndonna Marrast, Rehana Rasul, Ratnam Srivastava, Jeffrey Kuvin, Robert Roswell, Joseph Conigliaro, Eun Ji Kim

**Affiliations:** 1 Division of Cardiology, Rutgers Robert Wood Johnson School of Medicine, New Brunswick, New Jersey, United States of America; 2 Division of General Internal Medicine, Donald and Barbara Zucker School of Medicine at Hofstra/Northwell, Lake Success, New York, United States of America; 3 Department of Epidemiology and Biostatistics, Graduate School of Public Health and Health Policy, City University of New York, New York, New York, United States of America; 4 Department of Cardiology, Zucker School of Medicine at Hofstra/Northwell, Manhasset, New York, United States of America; 5 Lennox Hill Hospital, Zucker School of Medicine at Hofstra/Northwell, Hempstead, New York, United States of America; Dartmouth Health, UNITED STATES OF AMERICA

## Abstract

**Background:**

There are disparities in statin therapy for the secondary prevention of atherosclerotic cardiovascular disease (ASCVD). The role of structural racism in this disparity has not been examined.

**Methods:**

This is a cross-sectional study of participants with ASCVD in the Medical Expenditure Panel Survey from 2014–2017. Mediation analysis is utilized to estimate the direct effect of race and indirect effect of financial resources, access to care, and quality of care on statin usage.

**Results:**

The proportion of participants using statins by race/ethnicity were 58.5% for non-Hispanic Whites, 45% for Hispanics, 48.6% for Blacks, 61.6% for Asians, and 46.8% for Others. Statin usage was lower for Hispanics (OR = 0.79, 95% confidence interval [0.65–0.96]) and Blacks (OR = 0.80 [0.66–0.95]) compared to Whites. Hispanic, Black, and Other participants with the same financial resources, access to care, and quality of care as White participants did not have significantly different statin usage compared to White participants (Hispanic: OR = 0.98 [0.79–1.13]; Black (OR = 0.88 [0.76–1.06], Other: OR 0.76, 95% CI [0.56–1.15]). Hispanic, Black, and Other participants had significantly lower statin usage than subjects of the same race but with financial resources, access to care, and quality of care observed in White subjects (Hispanic: OR = 0.83 [0.83–0.92]; Black: OR = 0.91[0.88–0.94]; Other: OR = 0.92 [0.87–0.98]).

**Discussion:**

The indirect effect of race and ethnicity on statin therapy are significant but the direct effect of race and ethnicity on statin therapy are insignificant among Blacks and Hispanics compared to non-Hispanic Whites. This suggests that racial disparities in statin therapy are mediated through inequitably distributed resources, suggestive of the impact of structural racism.

## Background

Cardiovascular diseases is the leading cause of death in the United States [1, 2]. The prevalence of ischemic heart disease is projected to rise among Black and Hispanic populations and fall in White populations, worsening disparities in cardiovascular health [3, 4]. The American College of Cardiology has recommended statin therapy for clinical atherosclerotic cardiovascular disease (ASCVD) [5]. However, there are racial and ethnic disparities in the utilization of statins, even after controlling for region, age, sex, income, education, and comorbidities [4, 6–9]. Sociodemographic characteristics, including quality of care, insurance, source of care, and cost of statins have been associated with racial and ethnic disparities in statin therapy [9–11].

Structural racism refers to the interrelated and mutually reinforcing racial inequalities across major societal institutions, including health, law, wealth, criminal justice, and culture [12, 13]. Without the theoretical framework of structural racism, measured differences across race confound the effects of ethnicity, culture, ancestry, and broad societal inequalities, leading to an ambiguous and unactionable understanding of the problem. Understood through the theory of structural racism, racial disparities instantiate the result of systemic and continued oppression of racial and ethnic minorities [14, 15]. Past studies have reported disparities in statin usage by itself or attributed differences in terms of health service availability, interpersonal bias by physicians, and individual mistrust by patients [16]. However, discussion of results have not connected disparities to systemic racism.

To examine structural racism as a mediator for racial and ethnic disparities in statin therapy, we used the Medical Expenditure Panel Survey (MEPS), which contains nationally representative data of community dwelling Amefricans. We selected variables that captured the systemic impact of structural racism on acquiring statin therapy. Using these variables, we conduct a mediation analysis to quantitatively test whether demographic variables, socioeconomic variables, measures of access to care, and the physician-patient interaction together significantly mediate the effect of race on statin usage. We hypothesize that the racial and ethnic disparities in statin therapy are mediated by these markers of structural racism.

## Methods

All data from the Medical Expenditure Panel Survey are publicly available through the Agency for Healthcare Research and Quality and can be accessed at https://meps.ahrq.gov/mepsweb/.

### Study population and survey years

We used MEPS, a nationally representative household survey of non-institutionalized Americans from 2014 through 2017. The survey contains information about respondent’s demographics, health, and medical expenditure where heads of household are interviewed about the members of their household. We included surveys from 2014 to 2017 because statins were first recommended for secondary prevention for ASCVD in 2013 and 2017 was the last year with complete data for the desired variables.

For this study, we selected patients who were 18 years or older and had atherosclerotic cardiovascular disease defined as reporting a history of angina, myocardial infarction, coronary heart disease, stroke, acute myocardial infarction, angina pectoris, other forms of chronic ischemic disease, pre-cerebral occlusion, cerebral artery occlusion, transient cerebral ischemia, and atherosclerosis [7].

### Study variables

The primary outcome of interest was reported statin usage. Household respondents were asked what prescription medications were taken by household members. Participants were reported as receiving statin therapy if the respondent reported that they were taking an HMG-Coa reductase inhibitor or anti-hyperlipidemic combinations. All anti-hyperlipidemic combinations in MEPS contain an hmg-coa reductase inhibitor [7]. Tabulations of all the above variables and missing data are tabulated and stratified by race.

We used respondents’ self-reported race and ethnicity to categorize subjects as non-Hispanic White, Hispanic, non-Hispanic Black, non-Hispanic Asian, and non-Hispanic other. These groups are referred to as Hispanic, Black, Asian, White, and Other.

It is challenging to conceptualize a reliable and comprehensive measure of the ways structural racism impacts an individual’s life. However, it is plausible to measure the impact of structural racism on a discrete outcome when there is a well-defined causal pathway. In the case of statin usage, the myriad impacts of structural racism funnel down to the final process of navigating the health system to obtain a prescription for one’s use. In this process, a patient must use their financial resources to access care from doctors and pharmacies to ultimately receive a certain quality of care. Therefore, we hypothesize that this final sequence of events, which bears the weight of a multitude of upstream societal influences, can be estimated through its associations with an individual’s resources, access to care, and quality of care.

Mediator variables for resources were insurance coverage (any private insurance, public insurance only, uninsured), income (<100% federal poverty line (FPL), 100–125% FPL, 125–200% FPL, 200–400% FPL, and > 400% FPL). Variables representing access to care included time to usual provider (less than 15 minutes, 15 to 30 minutes, 31 to 60 minutes, 61 to 90 minutes, and 91 to 120 minutes), whether subjects were unable to obtain necessary medical or pharmaceutical care respectively, whether subjects delayed necessary medical or pharmaceutical care respectively, and how many appointments they had with their usual provider in the last year. Variables that assessed the patient’s perceived quality of care included an overall rating of healthcare from their usual provider on a scale of 1 to 10 and whether the subject’s usual provider never, sometimes, usually, or always listened carefully, explained things easily, showed respect, and spent enough time.

### Statistical analysis

While accounting for survey design, we conducted factor analysis with diagonally weighted least squares and oblimin rotation on all effect mediators to reduce the dimensionality of the mediating variables from 13 to three variables. Factor analysis with more extracted variables were limited because of estimations with negative variances. This was acceptable because the primary motivation for the factor analysis was reducing dimensionality, not exploratory analysis. Factor loading, interpretation of the factors, and goodness of fit are reported.

We first conducted descriptive statistics by race/ethnicity. Then, mediation analysis by using the product method with multiple mediators [17]. First, each log-transformed factor was regressed over age, sex, and BMI to give three separate models predicting the mediating factors based on race and confounders. These coefficients estimate the difference in factors for each race when controlled for confounders. Second, the log odds of statin usage were regressed over all factors simultaneously, race, age, sex, and BMI. The coefficients for each factor estimate the change in statin usage while controlling for confounders and the other two mediating factors. This method accounts for confounding among mediators and avoids double-counting of effects. The unadjusted effects are obtained by regressing the log odds of statin usage over race, age, sex, and BMI. Confidence intervals are calculated using standard errors. The natural indirect effects (NIE) are calculated by summing the products of the coefficient for each factor in the regression predicting statin usage multiplied by the coefficient for race predicting that factor. The natural direct effect (NDE) is the coefficient for race in the regression over all extracted factors, race, age, sex, and BMI. Confidence intervals are calculated by nonparametric boot strapping.

Sensitivity analyses for missingness were performed by imputing extreme data for variables assessing financial resources, access to care, and quality of care. In the first analysis, all missing data was imputed as reflecting the worst possible social determinants of health (i.e. uninsured, long distances to doctors, never feeling respected by providers). In the second analysis, all missing data was imputed to reflect the best possible social determinants of health (i.e. private insurance, short distance to doctors, always feeling respected by providers). The study was reviewed by the Feinstein Institutes for Medical Research Internal Review Board. Statistical analysis was completed using R and the following packages: lavaan.survey, survey, tableone, svyglm.

## Results

There were 12,860 participants representing 25.1 million Americans with ASCVD (Table 1). The mean age was 65.7 years old, 46.4% were female, and the mean BMI was 29.1 kg/m^2^. Overall, 55.7% of participants with ASCVD reported statin use. The proportions of statin use were 58.5% for non-Hispanic Whites, 45% for Hispanics, 48.6% for non-Blacks, 61.6% for Asians, and 46.8% for Others. After adjusting for age, sex, and BMI, but not mediating factors, statin use was significantly lower for Hispanic (OR = 0.79, [95% CI 0.65–0.96]) and Black subjects (OR = 0.80, [95% CI 0.66–0.95]) compared to non-Hispanic Whites. Statin usage use was higher in Asian participants (OR = 1.57, 95% CI [1.10–2.22]) compared to non-Hispanic Whites.

**Table 1:** Demographics stratified by race with total column per 1,000.

	Overall	White	Hispanic	Black	Asian	Other
n	25137	18209	2570	2891	741	726
Age (mean (SD))	65.7 (14.1)	67.2 (13.6)	60.5 (15.2)	61.9 (14.2)	64.9 (14.9)	60.1 (12.8)
Female (%)	11672 (46.4)	8107 (44.5)	1354. (52.7)	1570 (54.3)	274 (37.0)	368 (50.7)
Insurance coverage (%)						
Any private	13393 (53.3)	10652 (58.5)	816. (31.7)	1230 (42.5)	348 (46.9)	348 (47.9)
Public only	10731 (42.7)	7062 (38.8)	1451 (56.4)	1526 (52.8)	368 (49.7)	323 (44.5)
Uninsured	1013 (4.0)	495 (2.7)	304 (11.8)	135 (4.7)	25 (3.4)	55 (7.5)
Income category (%)						
<100% FPL	4041 (16.1)	2355 (12.9)	647 (25.2)	743 (25.7)	125 (16.9)	17 (23.5)
100–125% FPL	1672 (6.6)	950 (5.2)	255 (9.9)	341 (11.8)	64 (8.7)	61 (8.4)
125–200% FPL	4249 (16.9)	3004 (16.5)	506 (19.7)	521 (18.0)	84 (11.3)	134 (18.5)
200–400% FPL	7060 (28.1)	5211 (28.6)	758 (29.5)	697 (24.1)	208 (28.0)	19 (25.5)
>400% FPL	8115 (32.3)	6689 (36.7)	403 (15.7)	589 (20.4)	260 (35.1)	175 (24.1)
Have usual care provider (%)						
Yes	22022 (87.6)	16138 (88.6)	2120 (82.5)	2472 (85.5)	643 (86.9)	647 (89.3)
No	2047 (8.1)	1315 (7.2)	350 (13.6)	280 (9.7)	47 (6.3)	57 (7.8)
Missing	1068 (4.2)	757 (4.2)	100 (3.9)	139 (4.8)	50 (6.8)	21 (2.9)
Time to usual care provider (%)						
Less than 15 minutes	9975 (39.7)	7499 (41.2)	918 (35.7)	1036 (35.8)	281 (37.9)	241 (33.2)
15 to 30 minutes	9294 (37.0)	6745 (37.0)	904 (35.2)	1104 (38.2)	265 (35.8)	276 (38.0)
31 to 60 minutes	2186 (8.7)	1523 (8.4)	223 (8.7)	273 (9.4)	66 (8.9)	100 (13.9)
61 to 90 minutes	314 (1.3)	221 (1.2)	35 (1.4)	17 (0.6)	18 (2.5)	23 (3.2)
91 to 120 minutes	86 (0.3)	49 (0.3)	19 (0.7)	7 (0.3)	8 (1.1)	2 (0.3)
More than 120 minutes	91 (0.4)	56 (0.3)	18 (0.7)	11 (0.4)	5 (0.7)	1 (0.2)
Missing	319 (12.7)	2116 (11.6)	453 (17.6)	442 (15.3)	97 (13.1)	82 (11.2)
Delayed in obtaining necessary medical care (%)						
Yes	1669 (6.6)	1223 (6.7)	128 (5.0)	214 (7.4)	38 (5.1)	67 (9.2)
No	22643 (90.1)	16378 (89.9)	2358 (91.7)	2588 (89.5)	680 (91.8)	639 (88.1)
Missing	825 (3.3)	609 (3.3)	85 (3.3)	89 (3.1)	23 (3.1)	20 (2.7)
Unable to obtain necessary medical care (%)						
Yes	1044 (4.2)	752 (4.1)	94 (3.7)	130 (4.5)	28 (3.8)	40 (5.5)
No	23266 (92.6)	16848 (92.5)	2389 (93.0)	2672 (92.4)	690 (93.1)	666 (91.8)
Missing	828 (3.3)	610 (3.4)	87 (3.4)	89 (3.1)	23 (3.1)	20 (2.7)
Delayed in obtaining pharmaceuticals (%)						
Yes	1738 (6.9)	1295 (7.1)	122 (4.7)	226 (7.8)	26 (3.6)	69 (9.5)
No	22586 (89.9)	16327 (89.7)	2362 (91.9)	2574 (89.0)	686 (92.5)	638 (87.9)
Missing	813 (3.2)	587 (3.2)	86 (3.3)	92 (3.2)	29 (3.9)	20 (2.7)
Unable to obtain pharmaceuticals (%)						
Yes	1033 (4.1)	729 (4.0)	110 (4.3)	137 (4.7)	15 (2.0)	43 (5.9)
No	23289 (92.6)	16895 (92.8)	2374 (92.4)	2658 (92.0)	697 (94.1)	664 (91.4)
Missing	816 (3.2)	586 (3.2)	86 (3.3)	96 (3.3)	29 (3.9)	20 (2.7)
Number of medical appointments in the last year (%)						
0	2566 (10.2)	1609 (8.8)	424 (16.5)	391 (13.5)	72 (9.7)	69 (9.5)
1	2079 (8.3)	1481 (8.1)	223 (8.7)	235 (8.1)	49 (6.6)	92 (12.7)
2	2925 (11.6)	2115 (11.6)	265 (10.3)	376 (13.0)	75 (10.2)	94 (12.9)
3	2601 (10.3)	1824 (10.0)	271 (10.6)	348 (12.1)	72 (9.8)	84 (11.6)
4	2800 (11.1)	2077 (11.4)	254 (9.9)	322 (11.1)	94 (12.7)	54 (7.4)
5 to 9	4595 (18.3)	3531 (19.4)	373 (14.5)	430 (14.9)	126 (16.9)	135 (18.6)
10 or more	3519 (14.0)	2740 (15.0)	295 (11.5)	300 (10.4)	94 (12.6)	91 (12.5)
Missing	4053 (16.1)	2833 (15.6)	465 (18.1)	489 (16.9)	159 (21.5)	107 (14.7)
How often providers listen carefully (%)						
Never	186 (0.7)	137 (0.8)	14 (0.6)	28 (1.0)	4 (0.5)	3 (0.4)
Sometimes	1338 (5.3)	926 (5.1)	126 (4.9)	170 (5.9)	40 (5.4)	76 (10.5)
Usually	5384 (21.4)	4240 (23.3)	387 (15.0)	474 (16.4)	156 (21.0)	128 (17.6)
Always	11956 (47.6)	8736 (48.0)	1175 (45.7)	1368 (47.3)	321 (43.4)	355 (49.0)
Missing	6274 (25.0)	4171 (22.9)	868 (33.8)	851 (29.4)	221 (29.8)	163 (22.5)
How often providers explained things in a way that was easy to understand (%)						
Never	179 (0.7)	115 (0.6)	31 (1.2)	25 (0.9)	5 (0.7)	3 (0.4)
Sometimes	1180 (4.7)	774 (4.3)	132 (5.1)	156 (5.4)	60 (8.0)	59 (8.1)
Usually	6136 (24.4)	4857 (26.7)	439 (17.1)	522 (18.1)	169 (22.8)	149 (20.5)
Always	11424 (45.4)	8308 (45.6)	1117 (43.5)	1355 (46.9)	289 (39.0)	355 (48.9)
Missing	6218 (24.7)	4156 (22.8)	851 (33.1)	832 (28.8)	219 (29.5)	160 (22.1)
How often providers showed respect for what you had to say (%)						
Never	146 (0.6)	103 (0.6)	18 (0.7)	13 (0.4)	6 (0.8)	6 (0.8)
Sometimes	1138 (4.5)	781 (4.3)	114 (4.4)	137 (4.7)	45 (6.1)	61 (8.3)
Usually	4862 (19.3)	3818 (21.0)	352 (13.7)	439 (15.2)	114 (15.4)	138 (19.1)
Always	12773 (50.8)	9347 (51.3)	1235 (48.1)	1472 (50.9)	358 (48.4)	360 (49.7)
Missing	6219 (24.7)	4161 (22.8)	851 (33.1)	830 (28.7)	217 (29.3)	160 (22.1)
How often health providers spent enough time with you (%)						
Never	252 (1.0)	170 (0.9)	35 (1.4)	20 (0.7)	6 (0.8)	21 (2.9)
Sometimes	1614 (6.4)	1116 (6.1)	164 (6.4)	208 (7.2)	55 (7.4)	71 (9.8)
Usually	6525 (26.0)	5050 (27.7)	505 (19.6)	615 (21.3)	202 (27.2)	153 (21.1)
Always	10499 (41.8)	7700 (42.3)	1017 (39.6)	1205 (41.7)	261 (35.2)	317 (43.6)
Missing	6248 (24.9)	4175 (22.9)	849 (33.0)	843 (29.2)	217 (29.3)	164.601.1 (22.5)
1 to 10 rating on quality of care from providers (%)	8.3 (1.8)	8.4 (1.8)	8.5 (1.9)	8.0 (2.1)	8.0 (1.9)	8.0 (2.2)
Body mass index (mean (SD))	29.1 (6.9)	28.9 (6.7)	29.5 (6.6)	30.5 (8.1)	25.0 (4.7)	30.1 (7.3)
Prescribed statin (%)	14001 (55.7)	10644 (58.5)	1157 (45.0)	1404 (48.6)	456 (61.6)	340 (46.8)

Factor analysis was conducted to reduce dimensionality and yielded three factors that were categorized as financial resources, access to care, and quality of care (Table 2). The time it takes to travel to one’s usual provider and the number of appointments in the past year did not load significantly on to any factor. Insurance and income were loaded to financial resources. Delay and inability to access medical care and delay and inability to access pharmaceutical care were loaded to access to care. Overall rating of the quality of care, and measures of how often providers listened, explained, showed respect, and gave appropriate time were loaded to quality of care. The factor analysis reduced dimensionality while maintaining a good fit to the data (Root Mean Square Error of Approximation (RMSEA) = 0.031, Comparative Fit Index (CFI) = 0.996). For financial resources, access to care, and quality of care, the proportions of total variance were 7%, 8%, and 40% respectively. Factor analysis with more factors were invalid due to negative variance.

**Table 2:** Factor loading for variables representing financial resources, access to care, and quality of care.

	Financial resources	Access to care	Quality of care
Insurance	-0.61	-0.02	0.00
Income	0.74	-0.01	0.00
Time to usual provider	-0.08	-0.03	-0.01
Delay in accessing necessary medical care	-0.02	0.53	0.04
Unable to access necessary medical care	0.02	0.54	0.00
Delay in accessing pharmaceuticals	-0.01	0.49	-0.02
Unable to access pharmaceuticals	0.00	0.50	-0.03
Number of appointments with provider in the last year	-0.06	-0.12	-0.05
How often providers listened	-0.01	0.00	0.83
How often providers explained	0.01	-0.03	0.75
How often providers showed respect	0.00	-0.01	0.84
How often providers gave appropriate time	-0.01	0.01	0.79
Rating of providers from 1–10	0.05	0.10	0.59

Highlighted cells indicate variables that contribute to respective factors given absolute values greater than 0.2.

In mediation analysis, the natural indirect and direct effects varied by different race and ethnicity (Table 3). For Hispanic, Black, and Other subjects the direct effect of race on statin usage was insignificant, but the natural indirect effect of race mediated by factors for resources, access, and care were significant. Regarding the natural direct effects, Hispanic, Black, and Other subjects with the same financial resources, access to care, and quality of care as White subjects did not have significantly different odds of statin usage compared to non-Hispanic Whites (Hispanic: OR = 0.98, 95% CI [0.79–1.13]; Black (OR = 0.88, 95% CI [0.76–1.06], Other: OR = 0.76, 95% CI [0.56–1.15]). Regarding the natural indirect effects, Hispanic, Black, and Other subjects had significantly lower odds of statin usage than subjects of the same race but with the same financial resources, access to care, and quality of care as White subjects (Hispanic: OR = 0.83, 95% CI [0.83–0.92]; Black: OR = 0.91, 95% CI [0.88–0.94]; Other: OR = 0.92, 95% CI [0.87–0.98]).

**Table 3:** Unadjusted effects, natural direct effects, and natural indirect effects for the association between race and statin prescription mediated by finances, access, and quality.

	Unadjusted Effects (OR [95% CI])*	Natural Direct Effects (OR [95% CI])*	Natural Indirect Effects (OR [95% CI])*
White (ref)	-	-	-
Hispanic	0.79 [0.65–0.96]	0.98 [0.79–1.13]	0.83 [0.83–0.92]
Black	0.80 [0.66–0.95]	0.88 [0.76–1.06]	0.91 [0.88–0.94]
Asian	1.57 [1.10–2.22]	1.74 [1.32–2.58]	0.96 [0.91–1.0]
Other	0.74 [0.46–1.20]	0.76 [0.56–1.15]	0.92 [0.87–0.98]

*Controlled for age, sex and body mass index

Overall, Asian participants had a higher odds of statin usage compared to Whites (OR 1.57, 95% CI [1.10–2.22]). For Asian participants, the direct effect of race on statin usage was statistically significant while the indirect effects of race mediated by resources, access, and quality of care were not statistically significant. Regarding the natural direct effects, Asians had a significantly higher odds of statin use compared to those Whites (OR = 1.74, 95% CI [1.32–2.58]) with the same mediating factors as non-Hispanic Whites. Regarding the natural indirect effects, Asian subjects had significantly lower odds of statin usage compared to other Asian subjects with the same mediating factors as White subjects. Sensitivity analyses for missing data demonstrated similar results.

## Discussion

In an analysis of MEPS, Black and Hispanic participants with ASCVD have lower odds of statin usage than non-Hispanic White participants with ASCVD. Disparities in financial resources, access to care, and quality of care significantly mediate this difference in statin usage. Our study estimated the natural direct effects and natural indirect effects that mediate these disparities. The natural direct effects compare statin usage between White and Hispanic, Black, or Other subjects when the mediating factors are the same as White subjects. These effects were statistically insignificant. The natural indirect effects compare statin usage between subjects of the same racial or ethnic group but with either the financial resources, access to care, and quality of care as either White subjects or their own racial group. These effects were statistically significant. These findings suggest that societal disenfranchisement drives racial disparities in statin usage between White subjects and Black, Hispanic or Other subjects.

The present study addresses the intersection of structural racism and health. First, cultural preferences, patient preferences, health literacy, and distrust of health systems do not appear to mediate disparities in statin usage to the extent that they are independent from commensurate societal disparities in our analysis. If these types of factors drove disparities independent of structural inequalities, then we would have likely observed significant direct effects of race. Second, care should be taken in interpreting the results of this study. This racial disparity is mediated by financial resources, access to care, and quality of care, but this does not entail that racial disparities are identical to these factors nor can they be reduced to these factors. More likely, structural racism is a separate entity that acts through these disparities in the context of complex cultural, social, and economic history. It would be misguided to address racial disparities by intervening on disparities in financial resources, access, and quality agnostic to race. Third, this study suggests that systemic racism is implicated in a cornerstone of therapy for cardiovascular disease. It follows that any disparity that could be ameliorated by equitable statin therapy for secondary prevention implicates structural racism.

Structural racism in the United States manifests out of the mutually reinforcing disparities across sectors of society that advantage White people over others. One might consider anti-racist action at a provider, health systems, and societal level. At a provider level, we note that quality of care was measured by patient surveys on how well they felt they were treated by their provider. While the nature of confounding across our mediating factors prevents unbiased estimation of the proportion of this effect compared to others, this study implicates the patient’s experience and provider bias as a barrier to equitable care [18]. As this data is based on surveys of patients, provider bias that is independent of how a patient perceives their care are not accounted for in this study. This underscores the importance of adopting anti-racist principles in clinical practice. At a health systems level, access to care might be addressed through streamlining processes that remove barriers to care, whether those are bureaucratic tasks that overwhelm patients, financial barriers to obtaining medications and care, or logistics of coming to appointments. At a societal level, local and national professional societies are well positioned to initiate anti-racist policies in alliance with sectors outside of healthcare.

The present study demonstrates one methodologic approach to estimating mediation by structural racism. Generally, estimating the impact of broad societal inequalities is difficult because of the concern for confounding between mediators and the wide breadth of significant covariates. Given that structural racism encompasses all facets of society, it might seem that all facets of society must be accounted for and anything short of this suffers unmeasured confounders and biased estimates. However, if there is a well-defined, concrete understanding of the immediate process leading to a particular outcome and how socioeconomic forces are implicated, then the most proximal causal measures of disparate access to resources are reasonable markers of the broader, upstream societal factors. For example, the current study organizes the selection of variables based on the concrete mechanism of obtaining a statin. While every facet of society may influence the disparity in statins, at the end of the causal chain patients obtain statins by leveraging financial resources to access a certain quality of care. Therefore, estimating financial resources, access to care, and quality of care conceptually stand in for the broader societal disparities.

The study also suggests that Asian participants have higher odds of statin therapy compared to White participants. Examination of statin use among Asian Americans has been limited [19, 20]. This disparity between Asian and White participants is not mediated by the selected markers of societal disenfranchisement. In fact, the estimated natural indirect effects for Asian participants decrease the odds of statin usage though without reaching statistical significance. To the extent that differences in access to care, quality of care, and financial resources correlate with systemic disadvantages in acquiring appropriate statin therapy, the higher odds of statin usage in Asian participants compared to White participants are not mediated by a structural advantage in American society. In our study, disparities that advantage White subjects were mediated by measures of structural racism and those that advantage Asian subjects were not. This is consistent with previous work on the conceptualization of race to denote master status and societal position of White people above others [15].

This study shows that there is a large gap in statin usage among patients with ASCVD. Overall, we estimate that slightly less than half of the United States population with atherosclerotic cardiovascular disease are receiving appropriate lipid lowering therapy. This is a notable, and impactful gap in care consistent with prior research [21].

There are several limitations. While the sampled population is generalizable to the broader population of the United States, findings about the racial disparity in statin usage cannot be readily generalized to all other racial disparities. We hypothesize that the significance of direct effects of race and indirect effects mediated by markers of systemic racism will vary across different disparities. For example, disparities that relate to the distribution of pathogenic genetic mutations associated with race may be more likely mediated through direct effects of race. Meanwhile, those that implicate allocation of resources and services may be more likely to be mediated by markers of systemic racism. The data is limited by a lack of Native-American populations and missing ethnic backgrounds of Asian Americans. We also could not include education due to missing data. The role of education should be investigated in future research. The survey is also noted to have a low response rate, however, with purposive sampling and weights, we believe that this data is the best representative data for this study.

The present study indicates that financial resources, access to care, and quality of care are significant mediators of racial and ethnic disparities in a cornerstone of therapy for ASCVD. Racial disparities in financial resources, access to care, and quality of care represent a manifestation of systemic racism. Therefore, race is only a factor in statin usage for secondary prevention in so far as systemic racism is a factor. This serves as quantitative evidence that corroborates broader recognition of the role of systemic racism in disparities in cardiovascular outcomes [22].
